# The impact of COVID-19 in the attendance of patients to the otolaryngology clinic: a retrospective review

**DOI:** 10.1186/s43163-021-00147-0

**Published:** 2021-08-09

**Authors:** Kanachai Boonpiraks, Yanin Nawachartkosit, Dhave Setabutr

**Affiliations:** 1 Faculty of Medicine, Thammasat University, Pathum Thani, Thailand; 2 Department of Otolaryngology, Chulabhorn International College of Medicine, Thammasat University, Thammasat University Hospital, Pathum Thani, Thailand

**Keywords:** COVID-19, Otolaryngology, Head and Neck, Outpatient, Patient volume, Epidemiology

## Abstract

**Background:**

To evaluate the impact of the COVID-19 outbreak on patient management at an Otolaryngology Head and Neck Surgery Department at a tertiary care center in Southeast Asia. This is a retrospective review. Patient load and diagnosis at the Outpatient Division of the Otolaryngology Head and Neck Surgery Department were reviewed at the height of the initial wave of the COVID-19 pandemic. Patient-specific data was then compared during the same timeframe one year prior. Patients were then grouped into an additional subspecialty subgroup based upon their diagnosis. Descriptive statistics were analyzed.

**Results:**

A total of 819 cases were identified in 2019 during the study period. At the peak of the first wave, cases fell to 483, constituting a 41% decrease between the years (*p* value = 0.083). The largest decrease was in Otology cases with a drop by 53% (*p* value = 0.047), with the smallest decrease noted in General cases. Laryngology visits overall showed an increase in cases by 41.7%. Moreover, new visits decreased by 35.5%, with the largest decrease in new Laryngology visits and new Head and Neck Oncology cases. New visits for general issues had the smallest drop in patients, decreasing by only 21% (*p* value = 0.006)

**Conclusions:**

The COVID-19 pandemic caused a significant decrease in overall cases in the Otolaryngology Head and Neck Surgery outpatient department. Thus, in anticipation of future outbreaks, interventions may be tailored according to these trends.

## Background

The novel coronavirus has placed a huge impact on the public across the globe. Initially identified in Wuhan City, Hubei Province, China, it has spread rapidly becoming the first pandemic in decades [1]. The initial case confirmed in Thailand was on January 13, 2020, becoming the first country outside China to report a positive Coronavirus disease (COVID-19) case [2]. The World Health Organization (WHO) would later designate COVID-19 as a Public Health Emergency of International Concern (PHEIC) [3]. Thailand reached its initial peak during its first wave with a maximum of 252 new cases a day on March 29, 2020. Thailand would peak again on August 4, 2021, during its fourth wave, with a total of 20,200 new cases [4]. In the initial country’s response, early screening and management constituted by the Ministry of Public Health, through cooperation from Rapid Response Teams, Village Health Volunteers, hospital professionals, and several public services, would allow for effective tracking of social contacts. Thailand would undergo a complete lockdown by closing all of its international borders on April 3, 2020, to mitigate further migration of the disease [5]. Public campaigning on protective measures such as social distancing, mask wearing, and routine handwashing was emphasized. Thailand would then gain recognition from the WHO on its relative sustained control [6].

Even so, this emerging virus posed a heightened threat towards healthcare workers, specifically in the field of Otolaryngology, who are routinely exposed to secretions during patient encounters. With many aerosolizing procedures within the realm of Otolaryngology, Otolaryngologists would be highly susceptible compared to some of their counterparts in the hospital as COVID is less transmissible unless an aerosol-generating procedure is being brought out [7–9]. Along with many other large academic centers in the region, Thammasat University Hospital delivered an institutive response towards the management of COVID-19 in an effort to minimize personnel exposure to infected patients and to reduce contact and, thus, minimize possible transmission within the hospital. Correspondingly, the Department of Otolaryngology enacted special initiatives during the initial outbreak to correlate with limited personal protective equipment (PPE), which has also been similarly implemented in other healthcare centers [8, 10]. Therefore, we wanted to evaluate the impact these initiatives had on patient care and management when compared to the same time period one year prior. As the pandemic is still widely impacting the world, we hope this report could provide insight into future management as physicians and administrators do their best to provide utmost care during strenuous circumstances.

## Methods

This is a retrospective study comparing the patient volume in the outpatient department (OPD) during the initial COVID-19 wave at a tertiary Otolaryngology Department in the greater metro area around Bangkok, Thailand. Diagnosis and patient characteristics were compared for the same timeframe one year prior to the COVID-19 pandemic. This study was approved by the Research Ethics Committee, and conducted at Thammasat University Hospital, Rangsit, Thailand.

Thammasat University Hospital, established in 1988, is currently a tertiary and teaching hospital located in Pathum Thani province, Thailand. Since its establishment, the hospital has grown to provide healthcare services for over eleven million people annually and accommodate roughly 800 inpatient beds. The University Board then approved the opening of a Field Hospital on the campus in a modified dormitory to accommodate positive cases with less severe symptoms. That same Field Hospital remains open today at time of publication.

### Data collection

Data collection was made using the electronic hospital database. Records of all patients visiting the Otolaryngology outpatient department between March 23 to April 3, 2020, were collected. Data from the same timeframe was reviewed for the year 2019. Patient’s demographic data was reviewed and categorized into sub-specialties based upon their diagnosis of record. Patients with incomplete data were excluded from the analysis.

### Data analysis and statistical test

Collected data was categorized into follow-up and new patient encounters. The patient’s diagnosis was then separated into one of six subgroups: General, Pediatric, Otology, Rhinology, Laryngology, and Head and Neck. Given that all of our fellowship-trained staff also evaluate and treat General patients, we chose to only focus on the diagnosis of the patient as opposed to the provider. Criteria for the subcategories are listed in Table 1. The statistical analysis used in this study included descriptive statistics to describe the change in patient volume and disease incidences over the COVID-19 outbreak period, and the same time the year prior. The change in incidence of cases in each Otolaryngology subspecialty and the nature of each case (follow-up versus new cases) were also reviewed. Each year’s data were compared using a chi-square test, with a *p* value of less than 0.05 considered significant. Statistical analysis was performed through the Stata 14 for Windows (StataCorp LLC, College Station, Texas, USA).

**Table 1:** Criteria for the Subcategories for patients who presented at the outpatient Otolaryngology department

Subcategories	Inclusion criteria
General subgroup	Patients who presented with a common ailment in Otolaryngology including: rhinitis, acute sinusitis, impacted cerumen, upper respiratory symptoms, infection of the ear canal, and abscess
Pediatrics subgroup	Patients under the age of 18 years of age
Otology subgroup	Patients who presented with hearing loss, dizziness, and other diseases such as: tympanic membrane perforation, benign paroxysmal positional vertigo (BPPV), and Meniere’s disease
Rhinology subgroup	Chronic sinusitis, nasal polyposis, nasal mass
Laryngology subgroup	Patients with hoarseness, laryngeal mass, and diseases such as but not limited to: vocal cord paralysis, laryngopharyngeal reflux, laryngitis, dysphagia and vocal cord dysfunction
Head and Neck subgroup	Previous diagnosis of malignancy or unknown neck mass

## Results

**Fig. 1:**
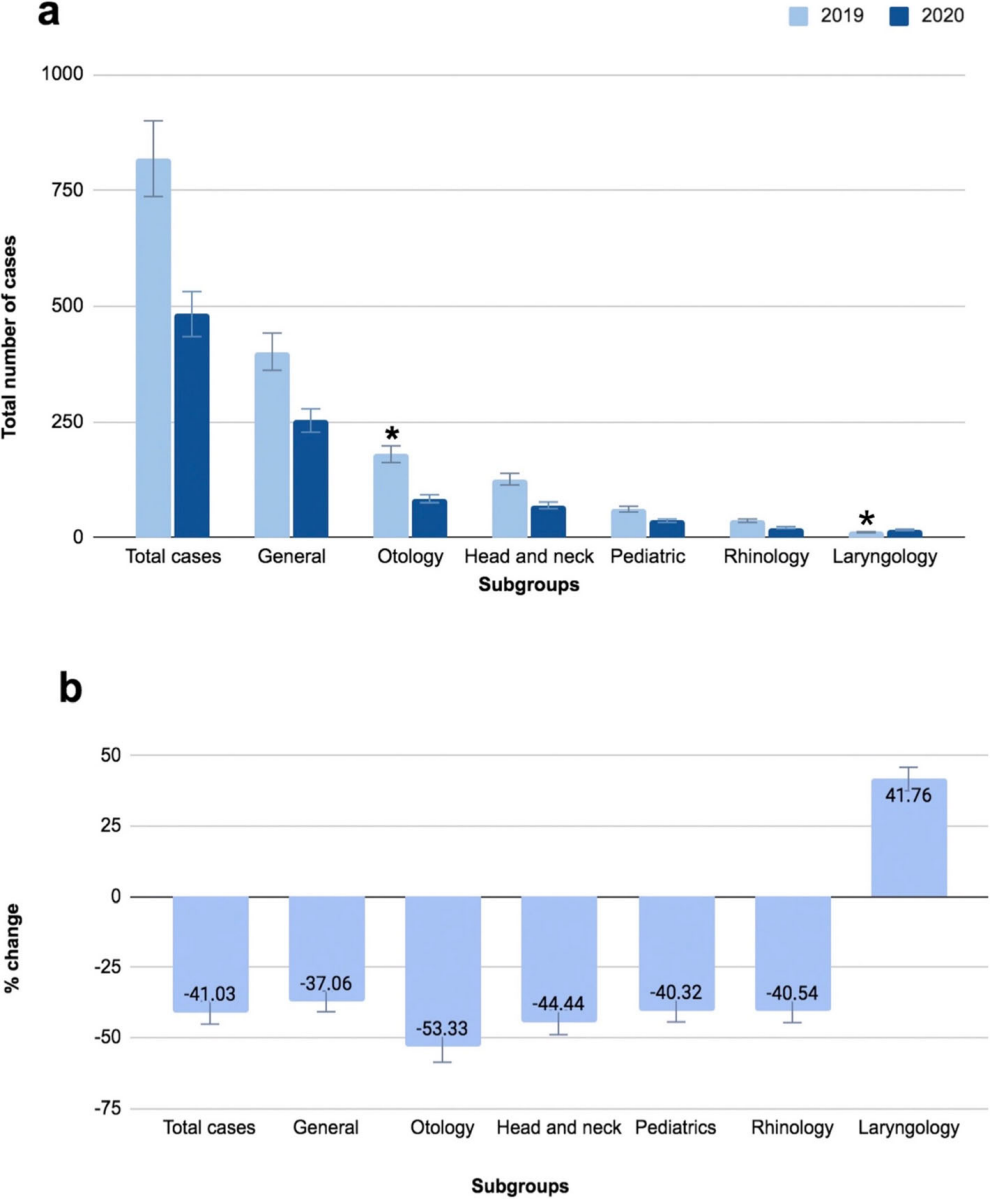
Comparison of the total number of outpatient visits between 2019 and 2020 in each subgroup: **a** total number of outpatient visits in each subgroup; **b** change in the percentage of visits *p < 0.05 (chi-square test)

Over the two study periods 1302 patient charts were reviewed. During 2019, a total of 819 cases were identified. The total number of cases fell to 483 in 2020. Table 2 lists a comparison amongst the patient’s age and sex compared before and during the initial COVID-19 outbreak. The median age of patients in 2019 was 53 years old (1–96 years old), while the median age in 2020 was 48 years old (0–96 years old). The ratio of male-to-female patients, when compared between the two-time frames, was similar. Table 3 and Fig. 1a demonstrate the comparison between the total number of outpatient cases in 2019 and 2020 within each subgroup. We noticed a 41% decrease in total number of cases (*p* value = 0.083). The largest decrease was in Otology cases with a drop by 53%, which was statistically significant (*p* value = 0.047). The smallest subset of patients to witness a decrease was in General cases as observed in Fig. 1b. Interestingly, the number of Laryngology cases increased by 41% and was statistically significant (*p* = 0.015). On the other hand, malignancy cases decreased by 6 cases, constituting a 13% drop (Table 4). Oncologic cases were all cases with a previous malignant diagnosis. Nevertheless, the proportion of oncologic cases to total outpatient cases in 2020 exceeded those in 2019.

**Table 2:** Patient’s demographic data

	2019	2020
Age: median (range)	53 (1–96)	48 (0–96)
Male: *N* (%)	341 (41.64%)	201 (41.6%)
Female: *N* (%)	478 (58.36%)	282 (58.39%)

**Table 3:** Comparison of the total number of outpatient visits between 2019 and 2020 in each Otolaryngology subgroup

	2019 *N* (%)	2020 *N* (%)	*P* value
Total case	819 (100)	483 (100)	0.083
Pediatric subgroup	62 (7.57)	37 (7.66)	0.953
Rhinology subgroup	37 (4.52)	22 (4.55)	0.975
Otology subgroup	180 (21.98)	84 (17.39)	0.047
Head and neck subgroup	126 (15.38)	70 (14.49)	0.664
General subgroup	402 (49.08)	253 (52.38)	0.250
Laryngology subgroup	12 (1.47)	17 (3.52)	0.015

**Table 4:** Comparison of malignancy cases in outpatient visits between 2019 and 2020

	2019 *N* (% of total outpatient visits)	2020 *N* (% of total outpatient visits)	Decrease in patients volume *N* (%)	*P* value
Diagnosis of malignancy	46 (5.62)	40 (8.28)	6 (13.04)	0.061

**Fig. 2:**
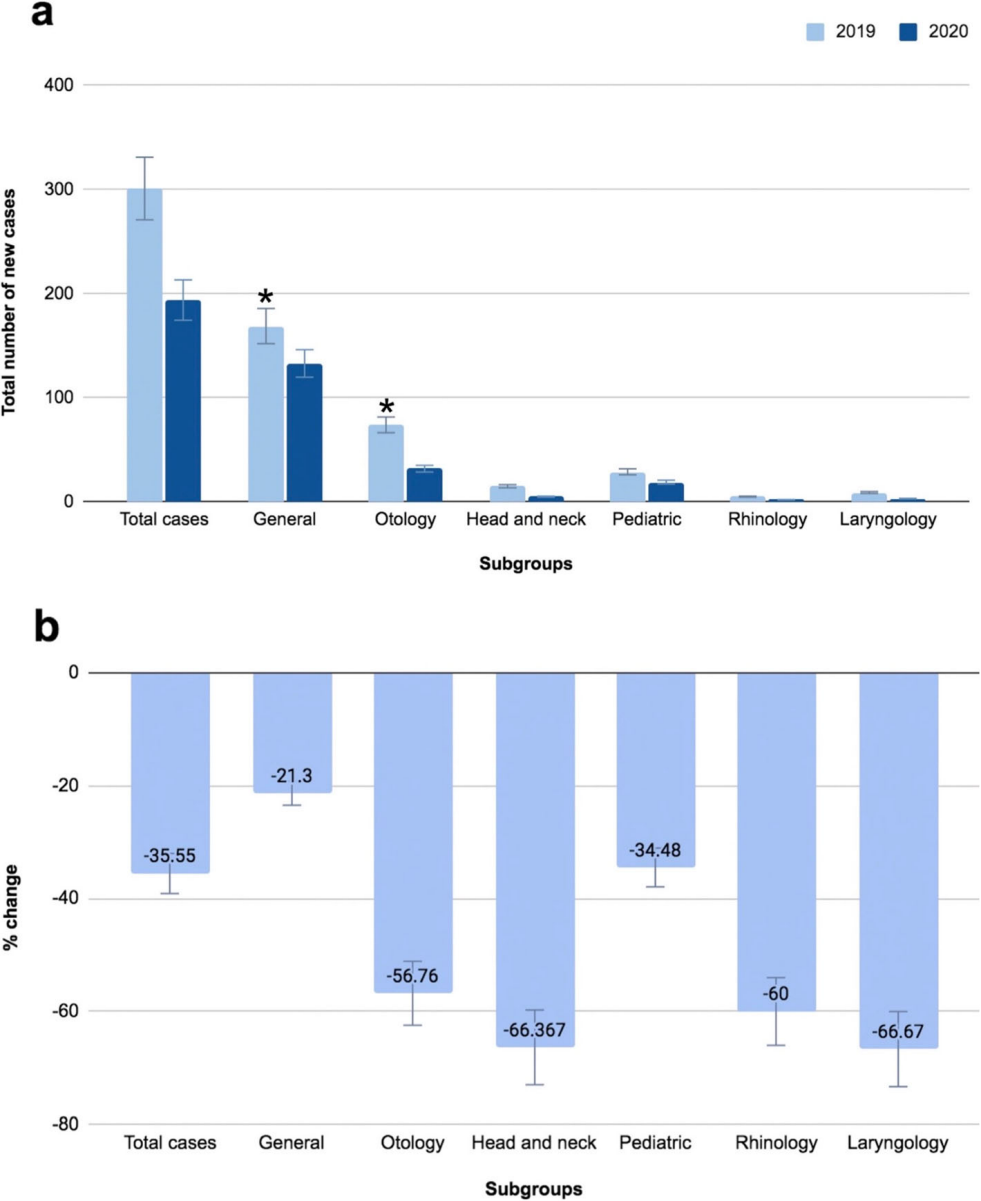
Comparison of outpatient visits between 2019 and 2020 in each Otolaryngology subgroup: **a** number of new outpatient visits in each Otolaryngology subgroup; **b** changes in the percentage of new visits *p < 0.05 (chi-square test)

Table 5 lists the change between the two years with regards to new cases, also displayed in Fig. 2a. New cases decreased during the same period from 2019 to 2020. Overall, a 35.5% decrease was noted. The largest decrease in new cases was noted in Laryngology cases, where we noticed a 66.7% drop, followed by Head and Neck oncology, with a 66.3% decrease. The General subgroup of new patients noticed the smallest drop in patients, decreasing by only 21.3% which was statistically significant, as pictured in Fig. 2b.

**Table 5:** Comparison of new outpatient visits between 2019 and 2020 in each Otolaryngology subgroup

	2019 *N* (%)	2020 *N* (%)	*P* value
Total case	301 (100)	194 (100)	0.087
Pediatric subgroup	29 (9.63)	19 (9.79)	0.953
Rhinology subgroup	5 (1.66)	2 (1.03)	0.562
Otology subgroup	74 (24.58)	32 (16.49)	0.032
Head and neck subgroup	15 (4.98)	5 (2.58)	0.184
General subgroup	169 (56.15)	133 (68.56)	0.006
Laryngology subgroup	9 (2.99)	3 (1.55)	0.308

## Discussion

With the first wave of COVID-19 in Thailand developing in early January 2020 along came the fear of the unknown and threat to the safety of the community. The COVID-19 virus posed a formidable task for hospitals as they functioned not only as a distributor of healthcare but also as a potential source of spread in the greater community. Thailand poised itself as a prepared leader in facing the pandemic [6]. Thammasat University Hospital became an essential part of our nation’s fight against the invisible threat. Cautious closure and cancellation of non-urgent visits were necessary to mitigate the initial spread of the virus [11, 12]. As a result, we felt it imperative to evaluate the overall impact those measures had on the Otolaryngology outpatient department. The initial wave resulted in a decrease in 41% of the total cases in patients presenting in the outpatient department, a trend also similarly established in other countries across the globe. Nonetheless, there have been reports of initial decline and subsequent normalization of outpatient cases to baseline within a matter of months, amidst the outbreak. This is believed to be pursued through abundant protective strategies and human resources, a measurement not yet available at our hospital [13]. Surprisingly, a study conducted in Singapore revealed an increase in outpatient attendances despite the positive trend in the pandemic curve. Such discrepancy may be due to the different nature of patients between the two populations [14]. Although no patient was denied access to healthcare be it through previously scheduled appointments or walk-ins, elective surgical cases were put on hold for approximately 6 weeks during the initial wave. Exceptions were made for those with a malignant diagnosis where the delay could further progress their disease [15, 16]. Therefore, the primary cause for the decrease in Head and Neck outpatient visits was secondary to the patient’s own decision-making. Alternatives were made available for patients who preferred to stay home opting to receive their routine home medications with drive-thru service or postal delivery, a management mechanism reported abroad [17, 18]. Being a large government-funded tertiary medical center, the hospital is known for prompting large crowds of patients at any given hour.

During the initial and subsequent outbreak, the hospital established a screening policy in the management and triage of patients who presented to the hospital inclusive of any of the following symptoms: fever, cough, sore throat, anosmia, or ageusia within the past 2 weeks. This protocol was also comparatively done in other healthcare systems [7, 8, 19]. Our patients were sent to an acute respiratory infection (ARI) screening clinic for assessment of COVID-19 risk and possible testing if criteria was met. Attentive triage of patients is a primary tool that has been widely used as a protective measurement against the transmission of the virus, especially when symptoms of COVID-19 are non-specific [13, 20, 21]. This became a defining factor for an overall decrease in General, Pediatric, and Rhinology cases overall. Different to many developed countries, specialist evaluation is not predicated on referrals from primary care physicians, even in the public government sector [14, 22]. Therefore, the aforementioned cases would be impacted greatly by the use of an ARI screening clinic. Additionally, Thailand’s experience with Telehealth is limited and has not been able to meet the needs of patients locally [23, 24].

Although the number of total cases showed a decrease across almost all subgroups, upon further inspection, it can be seen that oncologic patients only displayed a 13.04% decrease in the number of total cases, compared to the overall decrease amongst all patients at 41%. This signifies that although the COVID-19 had prevented many patients from visiting the hospital, patients with a malignant diagnosis continued their care uninterrupted during the pandemic. A study in Italy identified similar findings regarding the relative minimal decrease in oncologic patient volume [25]. Similar to most other countries with large outbreaks, reduction in operating room availability played a role [26]. Furthermore, a decrease in new cases within the head and neck oncology also puts a strain towards future management, as a delay in presentation or diagnosis of a potentially malignant condition can result in increased morbidity and mortality [16, 27]. Congruent to this statement, our Otolaryngology Department has the initiative to employ measurements that prioritize oncologic cases and Head and Neck subgroups so that their healthcare can be attended in person, while safety precautions are taken. Therefore, a virtual clinic appears to be an appropriate intervention, whether it is done by means of phone calls or video-assisted technology, to continue maintaining this level of care. Such an initiative should be proposed for cases with non-urgent presenting symptoms or those with a stable clinical status only. However, the withstanding limitations to telemedicine usage in Thailand, including patients without communication resources and lack of experience have put a barrier on the universality of this option. At press, virtual clinics have not been fully utilized at the hospital given parameters out of the scope of this paper; however, we have been able to initiate renewal of medications without need for doctor interaction. Therefore, as we endure our worst wave since the pandemic, virtual clinics should be considered again as a resource for especially those capable to utilize them. Diverting these cases to telemedicine will allow for safer overall care for those with emergent issues or elderly and disadvantaged patients who cannot partake in online treatment.

One of the limitations of this study is its narrow study population, as the participants were recruited from only one tertiary healthcare center. Therefore, the applicability of the results to other Otolaryngology departments elsewhere may be limited. Additionally, the study’s timeframe may have been affected by two holidays not present in 2020 as year to year changes in public holidays can occur per government decree. Thus, it can be inferred that the total number of cases may have been less regardless, possibly affecting the statistical significance in the total number of cases that decreased. It may be interesting to include the impact of surgical numbers in future studies, but that was out of our study’s scope.

## Conclusions

The COVID-19 pandemic has resulted in a marked decrease in all patients attending the outpatient Otolaryngology department. Certain conditions, such as oncologic cases, however, were able to show a relatively small decrease compared to its counterparts. The usage of designated virtual clinics holds a promising impact in the care of patients in the future. With the COVID-19 pandemic far from over and vaccinations not universally available, it will be important for countries with fragile healthcare structures to find ways to continue treating the public while also limiting spread of the disease.

## Data Availability

The raw data that support the findings of this study are available under request and with permission from Thammasat University Faculty of Medicine’s Ethics Committee.

## Abbreviations

ARI: Acute respiratory infection
BPPV: Benign paroxysmal positional vertigo
COVID-19: Coronavirus disease
OPD: Outpatient department
PHEIC: Public Health Emergency of International Concern
PPE: Personal protective equipment
WHO: World Health Organization
