# Correction to: The impact of COVID-19 in the attendance of patients to the otolaryngology clinic: a retrospective review

**DOI:** 10.1186/s43163-021-00153-2

**Published:** 2021-09-07

**Authors:** Kanachai Boonpiraks, Yanin Nawachartkosit, Dhave Setabutr

**Affiliations:** 1 Faculty of Medicine, Thammasat University, Pathum Thani, Thailand; 2 Department of Otolaryngology, Chulabhorn International College of Medicine, Thammasat University, Thammasat University Hospital, Pathum Thani, Thailand

**Correction to: Egypt J Otolaryngol 37, 82 (2021)**

**https://doi.org/10.1186/s43163-021-00147-0**

Following the publication of the original article [1], the authors identified errors in the Methods and Discussion section. The symbol * has been replaced with Thammasat.

The original article [1] has been corrected.
